# Physicochemical and biological similarity assessment of LBAL, a biosimilar to adalimumab reference product (Humira®)

**DOI:** 10.1080/19768354.2021.1943709

**Published:** 2021-06-23

**Authors:** Joon-Cheol Kwon, O Hwan Kwon, Rae Ung Jeong, Nayoun Kim, Seonah Song, Ilsub Choi, Juneok Lee, Takahiko Horiuchi

**Affiliations:** a Department of Biological Sciences, Korea Advanced Institute of Science and Technology (KAIST), Daejeon, Republic of Korea; b Life Science R&D campus, LG Science Park, LG Chem, Ltd., Seoul, Republic of Korea; c Department of Internal Medicine, Kyushu University Beppu Hospital, Beppu, Japan

**Keywords:** Adalimumab biosimilar, LBAL, analytical similarity assessment, physicochemical structure, biological function

## Abstract

LBAL was developed as an adalimumab (Humira®) biosimilar using Chinese hamster ovary cell lines. Comparable quality, safety, and efficacy between a biosimilar and its reference product should be ensured for regulatory approval. Here, we present the results of a comprehensive physicochemical and biological characterization between LBAL and Humira®. As physicochemical attributes, primary and higher-order structure, *N*-glycan profile, and disulfide linkage were investigated. Biological attributes were evaluated by target/receptor binding analysis and *in vitro*/*ex vivo* cell-based assays, which are linked to mechanisms of action. As a result, LBAL had the identical amino acid sequence, similar post-translational modifications and N-/C-terminal variants, and comparable primary, secondary, and tertiary structures and disulfide linkage profile. However, some differences in *N*-glycan profiles were observed. Biological activities, including tumor necrosis factor (TNF) binding, TNF-neutralization, apoptosis, Fc receptor binding, and complement-dependent cytotoxicity, were largely consistent. Despite a slightly lower antibody-dependent cellular cytotoxicity activity in LBAL, this difference was not significant under physiological conditions. As indicated, this extensive analytical characterization and functional comparison assessment showed that LBAL was similar to Humira®, with minor differences of no clinical relevance. Taken together, our comparative assessment of physicochemical and biological attributes demonstrated that LBAL is structurally and functionally very similar to Humira®, supporting the biosimilarity of clinical efficacy and safety.

## Introduction

A biosimilar is a biological product with high similarity in quality, potency, efficacy, and safety to a pre-approved reference product (RP) (European Medicines Agency [EMA] 2017; Kirchhoff et al. 2017; United States Food and Drug Administration [US FDA] 2017). With the impending patent expiry of high-priced original biologics, the development of biosimilars has been encouraged as a therapeutic alternative to offer a more affordable treatment option to patients and alleviate the financial burden on the healthcare system (McCamish and Woollett 2011; Kirchhoff et al. 2017). Due to the high complexity of biological products and inherent heterogeneity produced in living organisms, a biosimilar may inevitably have some minor differences from the RP. Nevertheless, these differences should be clinically irrelevant (EMA 2014a; US FDA 2015a).

Adalimumab (Humira®, AbbVie, Inc. 2021) is a recombinant human IgG1 monoclonal antibody (Ab) that prevents the interaction of tumor necrosis factor (TNF) with TNF receptors (TNFRs) by specifically binding to both soluble (sTNF) and transmembrane forms (tmTNF) of TNF. As a TNF blocker, adalimumab inhibits the biological effect of TNF, including the release of pro-inflammatory cytokines and apoptosis of the TNFR-bearing cells (Mitoma et al. 2008; Tracey et al. 2008; Horiuchi et al. 2010). This primary mechanism of action (MOA) is related to the treatment of inflammatory diseases, including rheumatoid arthritis, psoriasis, Crohn’s disease, and ulcerative colitis (Tracey et al. 2008; Olesen et al. 2016).

As an RP, Humira® (adalimumab) was first approved by the FDA in 2002 and by the EMA in 2003, respectively (Derbyshire 2019). ABP501 was developed by Amgen as the first biosimilar to adalimumab in 2016. Since then, numerous adalimumab biosimilars, including MSB11022, FKB327, PF-06410293, SB5, and GP2017, have been developed worldwide (Generics and Biosimilars Initiative [GaBI] 2021).

LBAL is another Humira® biosimilar developed and manufactured by LG Chem using Chinese hamster ovary (CHO) cells. During the LBAL development periods, the analytical (structural, physicochemical, and biological) quality attributes relevant to the MOA or with the potential to impact efficacy, potency, and safety were selected through the risk assessment according to the regulatory guidelines (US FDA 2019) and A-Mab case study (CMC-Biotech Working Group [CMC BWG] 2019). LBAL and Humira® were then evaluated by sensitive and orthogonal analytical approaches. The similarity ranges for selected quality attributes were provisionally determined by the characterization of tested Humira® lots.

Here, we present a comprehensive analytical characterization of the physicochemical and biological features of LBAL and Humira® to prove the biosimilarity. These similarity assessment results successfully show that LBAL is highly similar to Humira® at both the structural and functional levels, underpinning the biosimilarity of the following non-clinical and clinical trials.

## Materials and methods

### Materials

More than 20 lots of Humira® were procured from a pharmacy in Korea. Multiple batches of LBAL were produced by LG Chem (Korea). All chemical reagents, cell culture reagents, and the Biacore™ T200 instrument and its reagents were purchased from Sigma-Aldrich (St. Louis, USA), Gibco (Carlsbad, USA), and GE Healthcare (Uppsala, Sweden), respectively, unless otherwise noted. The recombinant proteins were purchased from R&D Systems (Minneapolis, USA), Sino Biological (Beijing, China), Quidel (San Diego, USA), and NIBSC (Hertfordshire, UK).

### Molecular mass analysis

The molecular masses of intact samples were measured using a quadrupole time-of-flight mass spectrometer (Q-TOF MS; Synapt G2, Waters, USA) coupled with an Acquity ultra-performance liquid chromatography (UPLC) instrument (Waters). The spectra data were deconvoluted by the BiopharmaLynx™ software (Waters). For molecular mass analysis of reduced and deglycosylated samples, the samples were pretreated with PNGase F (Roche, Germany) to remove *N*-linked glycans, and the disulfides were reduced with dithiothreitol.

### Reduced and non-reduced peptide analyses

Samples were denatured, then either reduced or non-reduced and alkylated with iodoacetic acid. The peptides digested by trypsin or Lys-C (Roche, Germany) were separated by UPLC. Eluting peaks were detected by UV (214 nm), and Q-TOF MS was used to determine the molecular mass. Disulfide bonds were analyzed by non-reduced tryptic peptide map analysis. The disulfide-linked peptides were identified by liquid chromatography-mass spectrometry (LC-MS) analysis.

### Glycan map analysis

The *N-*glycans released by PNGase F treatment were labeled with 2-aminobenzoic acid (2-AA, Merck), followed by hydrophilic interaction liquid chromatography (HILIC) purification. The 2-AA labeled *N*-glycans were subsequently separated by UPLC-fluorescence detection (FLD). The *N*-glycan structure of each peak was determined by mass spectrometry (MS) analysis. The MS/MS spectrum was obtained using the GlycoWorkbench software (developed by Alessio Ceroni).

### Higher-order structure analysis

A Chirascan™ Plus spectrophotometer (Applied Photophysics, UK) was used to obtain circular dichroism (CD) spectra. The absorption difference between left- and right-handed circularly polarized light in the far-UV (195–250 nm) and near-UV regions (250–320 nm) was measured. The measured CD signals were converted to mean residue ellipticity (MRE) using the CDNN software. Fourier transform infrared (FTIR) spectroscopy was performed using the Bruker Tensor™ II FTIR instrument equipped with an AquaSpec™ spectrophotometer. FTIR spectra were obtained in the range from 1800 to 1400 cm^−1^. The melting temperature (*T_m_*) was measured by differential scanning calorimetry (DSC). The thermogram was obtained using the DSC calorimeter (6300, TA Instruments).

### sTNF-α and fragment crystallizable receptor (FcR) binding by surface plasmon resonance (SPR) analysis

Amine-coupling was used to immobilize the target protein on a CM5 chip in a Biacore™ T200 instrument. For the sTNF-α binding assay, the fragment crystallizable (Fc) capture Ab was immobilized on the chip surface. The sample and TNF-α were sequentially injected, and the chip was regenerated. For Fc receptor (FcR)-binding, His-tagged FcγRI and FcγRIIb were captured on the chip surface via pre-immobilized anti-His capture Ab. FcγRIIa (R/H), FcγRIIIa (V/F), FcγRIIIb, and neonatal Fc receptor (FcRn) were directly immobilized on the chip. Serially diluted samples were injected, and an appropriate regeneration solution was used. The kinetics (*k*_a_, *k*_d_) and binding affinity (*K*_D_) for sTNF-α and FcγRI binding were determined from sensorgrams by 1:1 binding fit-model analysis using BIAevaluation™ software. Binding affinity for other FcRs was determined from sensorgrams by the steady-state response model.

### ELISA (enzyme-linked immunosorbent assay)

For tmTNF-α binding by cell-based ELISA, tmTNF-CHO cells were fixed with paraformaldehyde and blocked with casein. Samples were treated and incubated with anti-human IgG − HRP Ab (Sigma-Aldrich, USA), followed by treatment with the fluorogenic substrate (Thermo Fisher Scientific, USA). Fluorescence intensity was detected by a microplate reader. For C1q binding, the TNF-α-coated microplate was blocked with gelatin. Samples and human C1q were sequentially treated, followed by anti-C1q-HRP Ab (Bio-Rad, USA). ABTS substrate (Invitrogen, USA) was added, and absorbance was measured by a microplate reader at 415 nm.

### TNF-α neutralization assay

Samples were pre-incubated with TNF-α (NIBSC, UK) and actinomycin D (Sigma-Aldrich, USA), and then the mixtures were treated to L-929 cells (DSMZ, Germany). The cell viability was measured by the MTS assay (Promega, USA). The absorbance (490 − 650 nm) was measured by a microplate reader. The percentage of neutralization was calculated from the dose − response curve.

### Complement-dependent cytotoxicity (CDC)

Samples were incubated with the tmTNF-CHO cells in the presence of rabbit complement serum (Pel-Freez Biologicals, USA) at 37°C for 2 h to induce cell lysis. The cell viability was analyzed by the MTS assay, and the percentage of cytotoxicity was calculated from the dose–response curve.

### Ab-dependent cellular cytotoxicity (ADCC) using reporter assay and peripheral blood mononuclear cells (PBMCs)

The ADCC reporter assay kit (Promega) was used. tmTNF-CHO target cells were treated with samples and subsequently co-incubated with Jurkat reporter effector cells at 37°C for 6 h. Bio-Glo reagent was added, and the luminescent signal was measured on a luminometer (Molecular Devices, USA). PBMCs were isolated from healthy donors under the guidance of the institutional review board. For ADCC using PBMCs, tmTNF-CHO cells were treated with samples and PBMCs. After incubation, the luminescent cytotoxicity indicative of dead-cell protease activity was measured by the CytoTox-Glo® cytotoxicity assay (Promega). The relative activity was calculated from the dose–response curve.

### Apoptosis via reverse signaling

Apoptosis was analyzed using a MEBCYTO® apoptosis kit (MBL, USA). Samples were incubated with Jurkat-tmTNFα (Kyushu University, Japan) (Mitoma et al. 2008; Horiuchi et al. 2010; Ueda et al. 2013) for 48 h. The cells were stained with FITC–annexin V and propidium iodide (PI). The fraction of apoptotic cells was determined by flow cytometry. Apoptosis activity (%) was calculated by the percentage of apoptotic cells (annexin V-positive and PI-negative) (Bae et al. 2020; Kim et al. 2020).

### Data assessment and statistical analysis for biological activities

The relative binding or activity was calculated by the *K*_D_ or EC_50_ ratio of the test sample to that of the reference standard. Dose–response curves were fitted to a four-parameter logistic (4-PL) regression model using the parallel line analysis (PLA) software (Softmax® Pro; Molecular Devices, USA). The quality range (QR) for the similarity assessment is provisionally established as the mean ± 3 × standard deviation (SD) of RPs. The equivalence testing (*α* = 0.05) by the Minitab 18 software was applied to sTNF-α binding and neutralization. If the 90% confidence interval (CI) of the mean difference is within the equivalence acceptance criteria (EAC) defined by 1.5×SD of the Humira® lots, the analytical similarity is acceptable (Tsong et al. 2017).

## Results

The examined quality attributes included the primary structure, *N*-glycan profile, higher-order structure, and disulfide linkage, as well as the fragment antigen-binding (Fab)-, Fc-, and Fab-/Fc-related biological activities. A list of quality attributes, analytical methods used for similarity assessment, and summary results are described in Table 1. Considering that the comparability of Humira® has already been demonstrated comprehensively among Humira® sourced from different markets (European Union, Korea, and Japan), the comparison between the representative Humira® (Korea) and LBAL was addressed for the similarity assessment in this study.

**Table 1.** Summarized quality attributes with analytical methods and assessment results of the biosimilarity.

Category	Clinical relevance	Product quality attribute	Analytical methods	Assessment results
**Structural/physicochemical characterization**				
Primary Structure	Efficacy, Safety, Immunogenicity	Molecular weight	Intact and deglycosylated (reduced/non-reduced) by LC-MS	Highly similar
		Amino acid sequence	Peptide mapping by LC-MS/MS w/ tryptic & Lys-C digestions	Identical
		Post-translational modification: deamidation (Asn) & oxidation(Met)	Peptide mapping by LC-MS/MS	Highly similar
		N-/C-terminal variants		Similar
		Free sulfhydryl group	Measure-iT^TM^ Thiol Assay	Highly similar
Higher order Structure	Efficacy & Immunogenicity	Secondary structure	Far-UV CD	Highly similar
			FTIR spectroscopy	Highly similar
		Tertiary structure	Near-UV CD	Highly similar
			Fluorescence spectroscopy	Highly similar
		Thermodynamic stability	DSC	Highly similar
		Disulfide linkage mapping	Peptide mapping (non-reduced)	Highly similar
Carbohydrate Structure	Efficacy & Immunogenicity	*N*-linked glycosylation site	LC-MS/MS	Identical (Asn 301)
		*N*-glycan identification	LC-MS/MS	Highly similar
		*N*-glycan profile	2-AA labelling and HILIC-UPLC/MS	Similar in %[Gal]; slightly higher in %[Afucose]; lower in %[HM]; slightly higher in %[Sial]; **^†^**
Size heterogeneity (Purity/Impurity)	Efficacy & Immunogenicity	High molecular weight (HMW)	SE-HPLC, SEC/MALLS	Similar
	Efficacy	Low molecular weight (LMW)	CE-SDS (non-reduced/reduced)	Highly similar
Charge heterogeneity	Efficacy	Acidic and basic variants	CEX-HPLC, cIEF	Similar in acidic level; slightly lower basic level **^†^**
**Biological characterization**				
Fab-related biological activity	Efficacy	sTNF-α binding	SPR	Highly similar
		tmTNF-α binding	Cell-based ELISA	Highly similar
		sTNF-α neutralization	Cell-based assay (L929 cell, MTS assay)	Highly similar
		Apoptosis via reverse signaling	Flow cytometry (PI/Annexin V)	Highly similar
Fc-related biological activity	Efficacy	FcγRIa binding	SPR	Highly similar
		FcγRIIa (R131, H131) binding	SPR	Highly similar
		FcγRIIb binding	SPR	Highly similar
		FcγRIIIa (V158, F158) binding	SPR	Highly similar
		FcγRIIIb binding	SPR	Similar
	PK	FcRn binding	SPR	Highly similar
	Efficacy	C1q binding	ELISA	Highly similar
Fab- and Fc- related biological activity	Efficacy	CDC	Cell-based assay	Highly similar
		ADCC (reporter bioassay )	Reporter Assay	Slightly lower, but Similar
		ADCC (PBMC)	Cell-based assay	Highly similar

These methods and results are representative examples used for similarity assessment*.*

**^†^**not clinically meaningful

### Primary structure and *N*-glycan profiles

**Figure 1.**
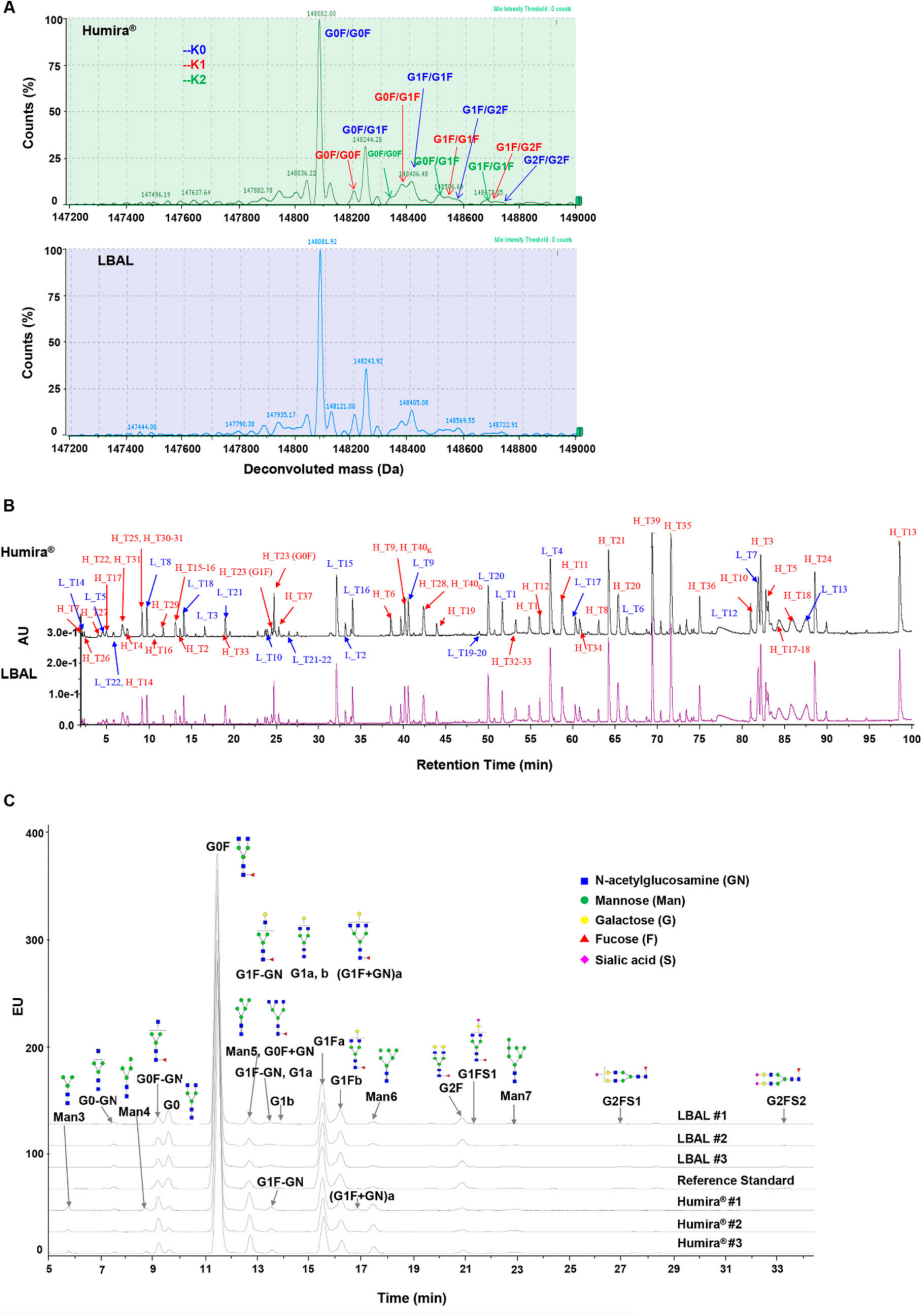
Primary structure: comparison of Humira® and LBAL. (**A**) Intact molecular mass profile by LC-MS, (**B**) UV chromatogram profiles of tryptic peptide map under reduced condition, (**C**) Fluorescence chromatogram profiles of 2-AA labeled *N*-glycan map by HILIC-UPLC/FLD. AU, absorbance unit; H, heavy chain; L, light chain; T, tryptic peptide; EU, fluorescence emission unit.

The validation of primary structure is crucial in defining the identity of LBAL as adalimumab. MS was used to determine the primary structure through intact mass analysis, reduced peptide mapping, and glycan map analysis (Figure 1). The peak profiles of the intact molecular mass between LBAL and Humira® were very similar (Figure 1A). The core fucosylated biantennary complex *N*-linked glycans with either zero, one, or two terminal galactose residues (i.e. G0F, G1F, and G2F, respectively) were observed as the prevalent species, consistent with the glycan map results (Figure 1C). Due to the heterogeneity of the attached glycans, multiple peaks were identified, and G0F/G0F was the most abundant glycoform for LBAL and Humira®. Likewise, C-terminal Lys variants containing zero, one, or two Lys residues (i.e. K0, K1, and K2, respectively) were similarly observed in the corresponding peaks. K0 type was the most abundant glycoform. The LC-UV tryptic peptide map of reduced LBAL and Humira® resulted in non-distinguishable UV chromatograms with high similarity in peak intensities and retention times (Figure 1B), inferring identical amino acid sequences.

The profiles of *N-*linked glycosylation were characterized by HILIC-UPLC/MS analysis of 2-AA-labeled *N*-glycans. The fluorescence chromatogram obtained from the 2-AA-labeled glycans showed that overall *N*-glycan profiles were similar between Humira® and LBAL, albeit some minor quantitative differences were observed in specific glycan species (Figure 1C). Neutral complex-type biantennary *N*-glycans (lacking sialic acid) were mainly observed. Afucosylated (Afucose) complex-type (lacking core fucose) and high mannose (HM)-type *N*-glycans were found at low abundance in both Humira® and LBAL. LBAL had relatively similar levels of %galactosylation but slightly higher levels of afucosylation and sialylation, with lower levels of the HM-type than Humira® (Supplementary Table S1). Therefore, the impact of these differences in *N*-glycan profiles on biological activity needs to be demonstrated in relation to the biological and functional attributes.

### Higher-order structure and disulfide linkage

Multiple biophysical methods were used to analyze the higher-order structure of LBAL and Humira®. The secondary structure was investigated by far-UV CD and FTIR spectroscopy, the tertiary structure by near-UV CD spectroscopy, and the thermal stability by DSC (Li et al. 2011; Thiagarajan et al. 2016).

**Figure 2.**
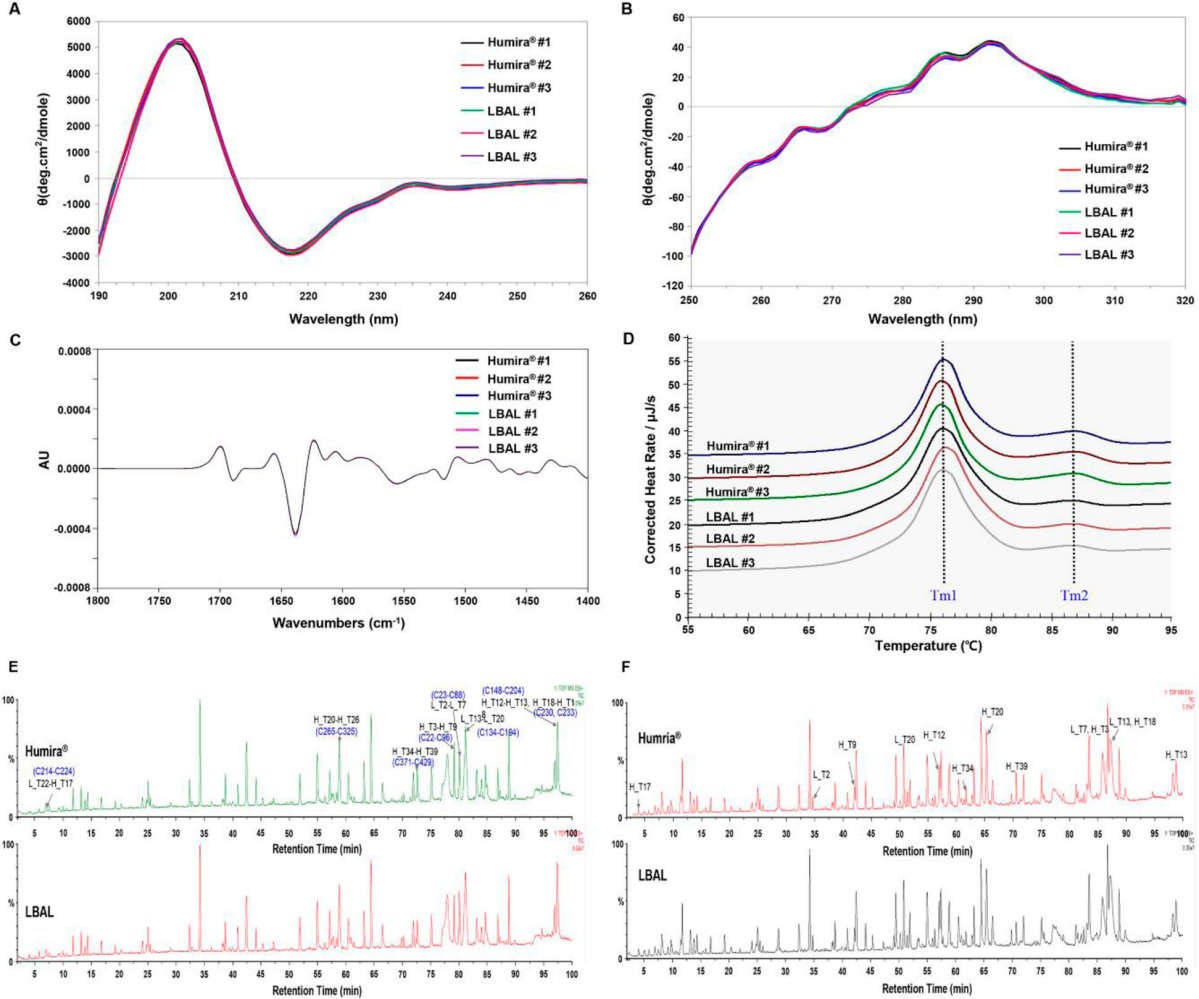
Higher-order structure: comparison of Humira® and LBAL. (**A**) Far-UV CD spectra for secondary structure, (**B**) Near-UV CD spectra for tertiary structure, (**C**) FTIR spectra of second-derivative for secondary structure, (**D**) DSC profile for thermal stability, and total ion chromatogram (TIC) profiles of tryptic peptide maps by LC-MS under non-reducing condition (**E**) and reducing condition (**F**) for disulfide linkage. AU, absorbance Unit; H, heavy chain; L, light chain; T, tryptic peptide.

The far-UV CD spectra of LBAL and Humira® were consistent. The pattern was typical of an Ab with a minimum at 217 nm and a maximum at 202 nm, indicating a secondary structure with dominant β-sheets (Figure 2A). The secondary structures were further confirmed by FTIR spectroscopy as an orthogonal method to CD. Comparisons of second-derivative amide I spectra revealed high similarity. A strong β-sheet band at ∼1639 and 1689 cm^−1^ (Figure 2C) was observed, indicating the presence of antiparallel β-sheet structures typical in Abs. The near-UV CD spectra were found to overlap indistinguishably, supporting comparable tertiary structures (Figure 2B). The spectra contained peak signals for aromatic amino acids (Trp: 255–275 nm; Tyr: 275–285 nm; Phe: 285–295 nm) and the disulfide bond (250–280 nm), reflecting the native folding structure. The overlaid DSC thermograms exhibited two endothermic thermal transitions with the *T_m_* values equivalent to the unfolding of the Fab/CH2 (*T_m_*_1_, 75.8–76.3°C) and CH3 (*T_m_*_2_, 86.6–87.1°C) domains (Figure 2D), supporting high resemblance in thermal stability.

Intra- and interchain disulfide bonds are crucial for Ab tertiary/quaternary structure formation (Liu and May 2012). Disulfide linkage mapping was performed by tryptic digestion without reduction. LC-MS profiles of the non-reduced peptides showed the expected disulfide linkage pattern in both Humira® and LBAL (Figure 2E). A total of 32 Cys residues (11 Cys in each heavy chain [HC] and 5 Cys in each light chain [LC]) were involved in 16 disulfide bonds (12 intrachain and 4 interchain), without free sulfhydryl groups. To identify the location of disulfide bonds, LC-MS analysis of the reduced peptides was also conducted. The presence of the expected Cys-containing peptides was consistent with the theoretical mass (Figure 2F and Supplementary Figure S1). The identified disulfide linkages are summarized in Table 2.

**Table 2.** Disulfide linkages of LBAL and Humira®.

Region	Type	Disulfide-Linked peptides	Cysteine site
H	Intra-chain	H_T3-H_T9	C22-C96
		H_T12-H_T13	C148-C204
		H_T20-H_T26	C265-C325
		H_T34-H_T39	C371-C429
L	Intra-chain	L_T2-L_T7	C23-C88
		L_T13-L_T20	C134-C194
H and L	Inter-chain	H_T17-L_T22	C224-C214
hinge region		H_T18=H_T18	C230-C230 = C233-C233

H: heavy chain, L: light chain, T: tryptic peptide, C: Cysteine (Cys).

### Fab-related biological activity

The primary MOA of adalimumab is the inhibition of the biological effects of TNF-α, which are directly mediated by the binding and neutralization of TNF-α through its Fab region. Four biological assays were conducted to assess the Fab-related biological activity of LBAL: two TNF-binding (sTNF-α and tmTNF-α), one sTNF-α neutralization, and one apoptosis.

**Figure 3.**
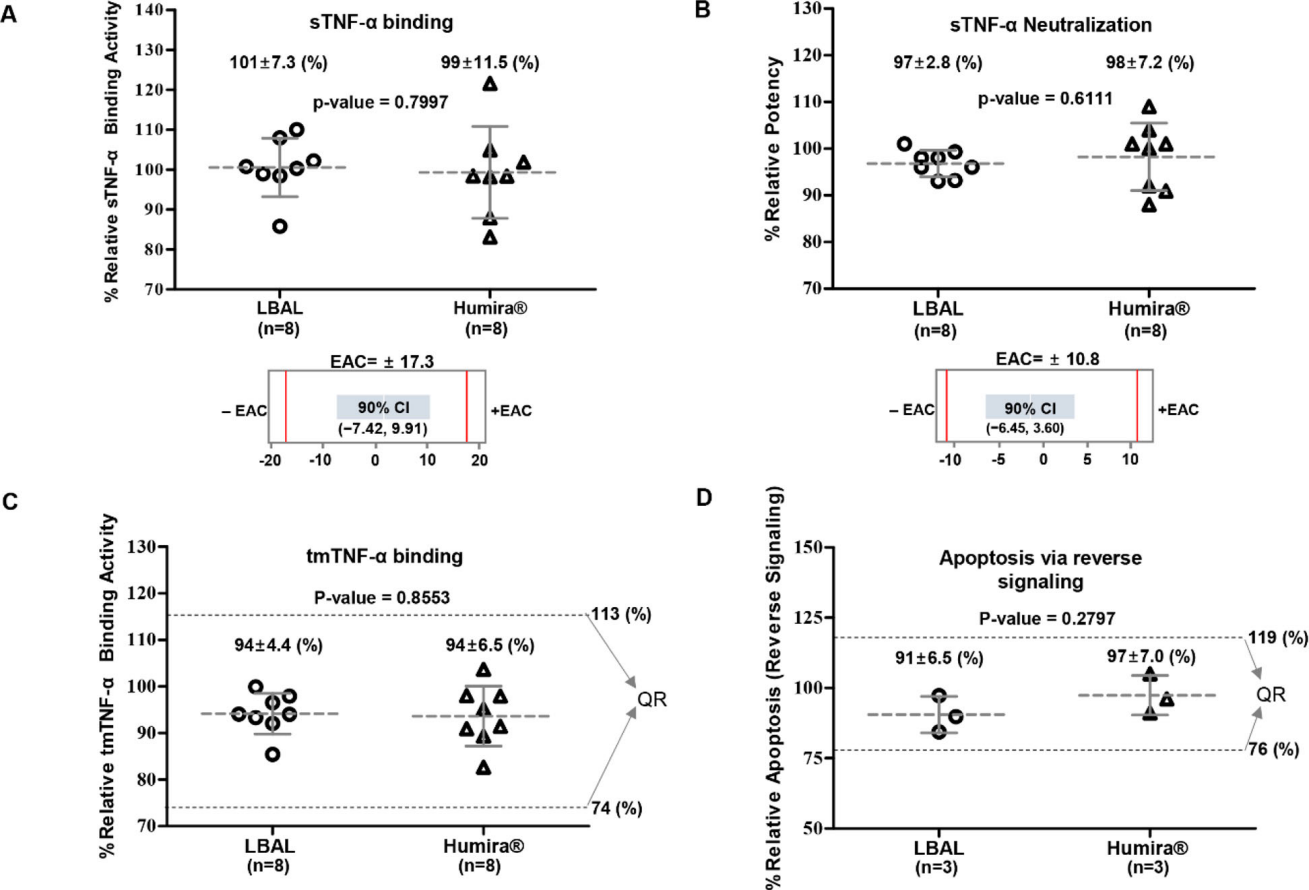
Similarity assessment of Fab-related biological activities: comparison of Humira® and LBAL. Dot plot profiles of (**A**) sTNF-α binding by SPR (**B**) sTNF-α neutralization in L-929 cells, (**C**) tmTNF-α binding by cell-based ELISA using tmTNF-CHO cells, (**D**) Apoptosis via reverse signaling on Jurkat-tmTNFα cells by flow cytometry analysis. Each point represents the mean value of a specific batch/lot. The gray dotted line for samples indicates the overall mean value. The error bar represents the standard deviation (SD). ‘n’ indicates the total number of lots tested. The mean value of each lot was determined by three independent experiments. The red line indicates −EAC and +EAC. Equivalence acceptance criteria (EAC). The scatter dot plots of (**A**) and (**B**) were plotted above corresponding equivalence test results showing the 90% confidence interval (CI) of the mean difference. Dashed lines (black) represent the similarity quality range (QR) (mean ± 3SD). Student’s *t*-test was used for statistical analysis. *p < 0.05, **p < 0.01, and ***p < 0.001.

Results of the relative sTNF-α-binding affinity measured by SPR exhibited statistically equivalent activity between LBAL and Humira®. The 90% CI for the difference of the means between LBAL and Humira® (−7.42, 9.91) was within the EAC of Humira® (±17.3%), demonstrating that LBAL and Humira® are highly similar in sTNF-α-binding activity (Figure 3A). The ability of LBAL to inhibit sTNF-α-induced cell death was assessed by sTNF-α neutralization using L929 cells. The potency of sTNF-α neutralization was equivalent. The 90% CI for the difference of the means (−6.45, 3.60) was within the EAC (±10.8%) (Figure 3B). The tmTNF-α-binding activity was evaluated by cell-based ELISA. The mean ranges of relative tmTNF-α-binding activity of LBAL (85–100%) were within the QR (74–113%), suggesting comparable tmTNF-α-binding activity (Figure 3C). The binding of tmTNF-α by adalimumab can result in apoptosis of tmTNF-α-bearing cells through reverse (outside-to-inside) signaling (Tracey et al. 2008; Olesen et al. 2016). Assessment of apoptosis in Jurkat-tmTNFα cells by fluorescence-activated cell sorting analysis showed the high similarity in apoptosis activity, supported by the mean ranges of the relative activity of LBAL (84–97%), which were within the QR (76–119%) (Figure 3D). Overall, LBAL showed highly similar Fab-related biological activities to Humira®. These findings are further detailed in Supplementary Table S2.

### Fc-related biological activity

**Figure 4.**
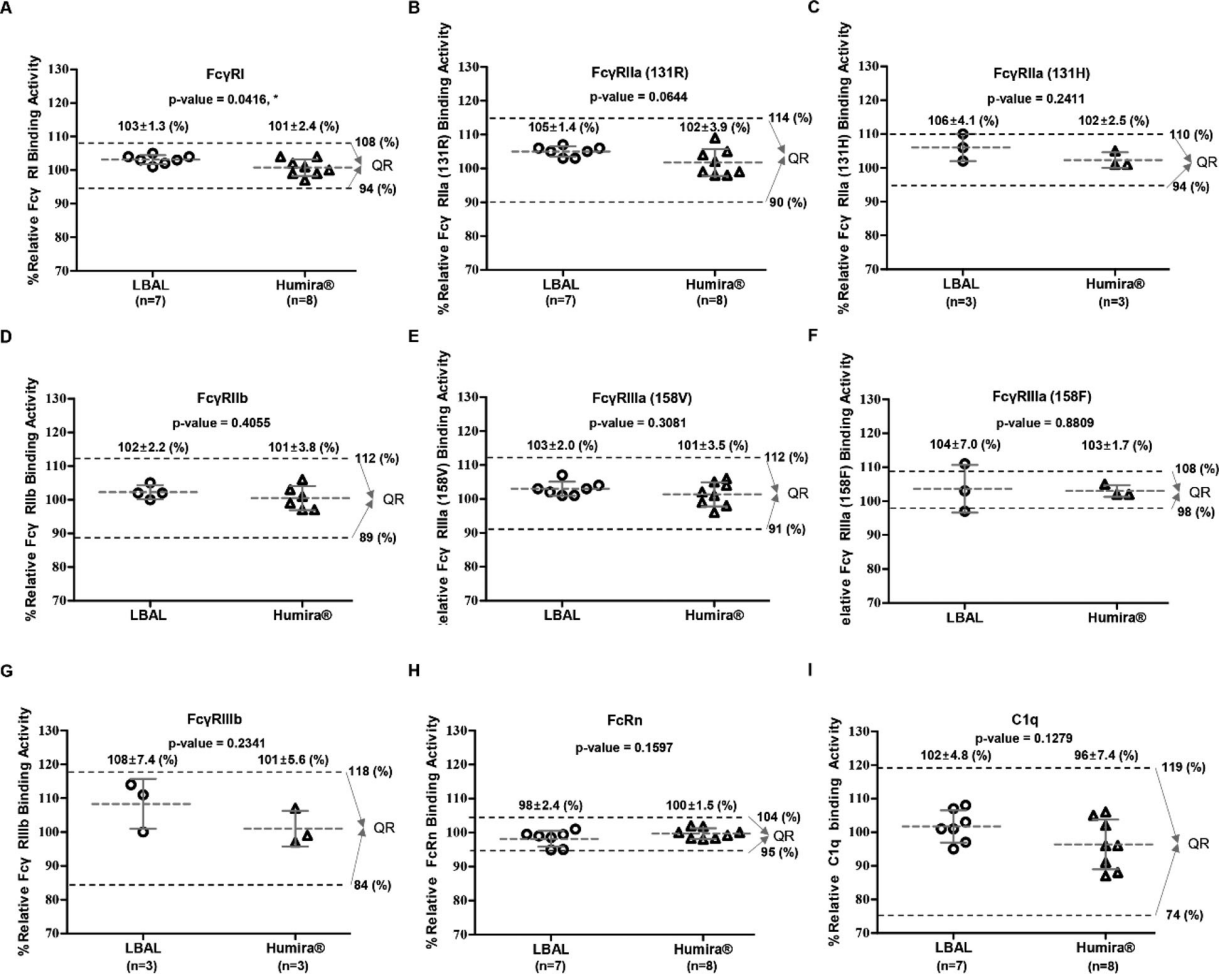
Similarity assessment of Fc-related biological activities: comparison of Humira® and LBAL. Scatter dot plots of relative binding activity for (**A**) FcγRI, (**B**) FcγRIIa (131R), (**C**) FcγRIIa (131H), (**D**) FcγRIIb, (**E**) FcγRIIIa (158 V), (**F**) FcγRIIIa (158F), (**G**) FcγRIIIb, (**H**) FcRn by SPR, and (**I**) C1q by ELISA. Dashed lines (black) represent the similarity quality range (QR) (mean ± 3SD). Student’s *t*-test was used for statistical analysis. *p < 0.05, **p < 0.01, and ***p < 0.001.

The Fc region of adalimumab interacts with a variety of FcγRs expressed on immune cells or soluble C1q in complement systems as well as FcRn on epithelial/endothelial cells, contributing to modulation of diverse immune responses, besides affecting its serum half-life/pharmacokinetics (Nimmerjahn and Ravetch 2008; Bruhns et al. 2009; Rosales and Uribe-Querol 2013; Ward et al. 2015; Hayes et al. 2016). To evaluate the Fc-related biological quality attributes, the binding affinity for various FcγRs (FcγRI, FcγRIIa (R/H) allotypes, FcγRIIb, FcγRIIIa (V/F) allotypes, and FcγRIIIb) was measured by SPR, and C1q-binding activity was analyzed by ELISA. As shown in Figure 4, the Fc (Figure 4A–H) and C1q binding activities (Figure 4I) were comparable between LBAL and Humira®. The mean ranges of relative binding activity of LBAL were within the QR for the corresponding biological activity. Although the mean ranges of relative FcγRIIIa (158F) of LBAL were slightly outside the QR of Humira® (Figure 4F), the mean relative binding activity per group was very similar. This observed difference (up to 1–3%) was not significant considering lot variations (*n* = 3), method variability, and the high sensitivity of SPR. Collectively, LBAL showed very similar Fc-related biological activities to Humira®. These findings are further detailed in Supplementary Table S3.

### Fab- and Fc-related biological activity (effector function)

The relative activities of CDC and ADCC were analyzed for Fab-/Fc-related biological activities of LBAL and Humira®. Adalimumab evokes CDC following lysis of tmTNFα-expressing target cells by activating the classical complement pathway via soluble C1q binding of its Fc portion (Bournazos and Ravetch 2017). ADCC occurs when adalimumab interacts with both tmTNF-α on target cells and FcγRIIIa on effector cells through its Fab and Fc domains, respectively (Mitoma et al. 2008; Tracey et al. 2008; Arora et al. 2009; Horiuchi et al. 2010).

**Figure 5.**
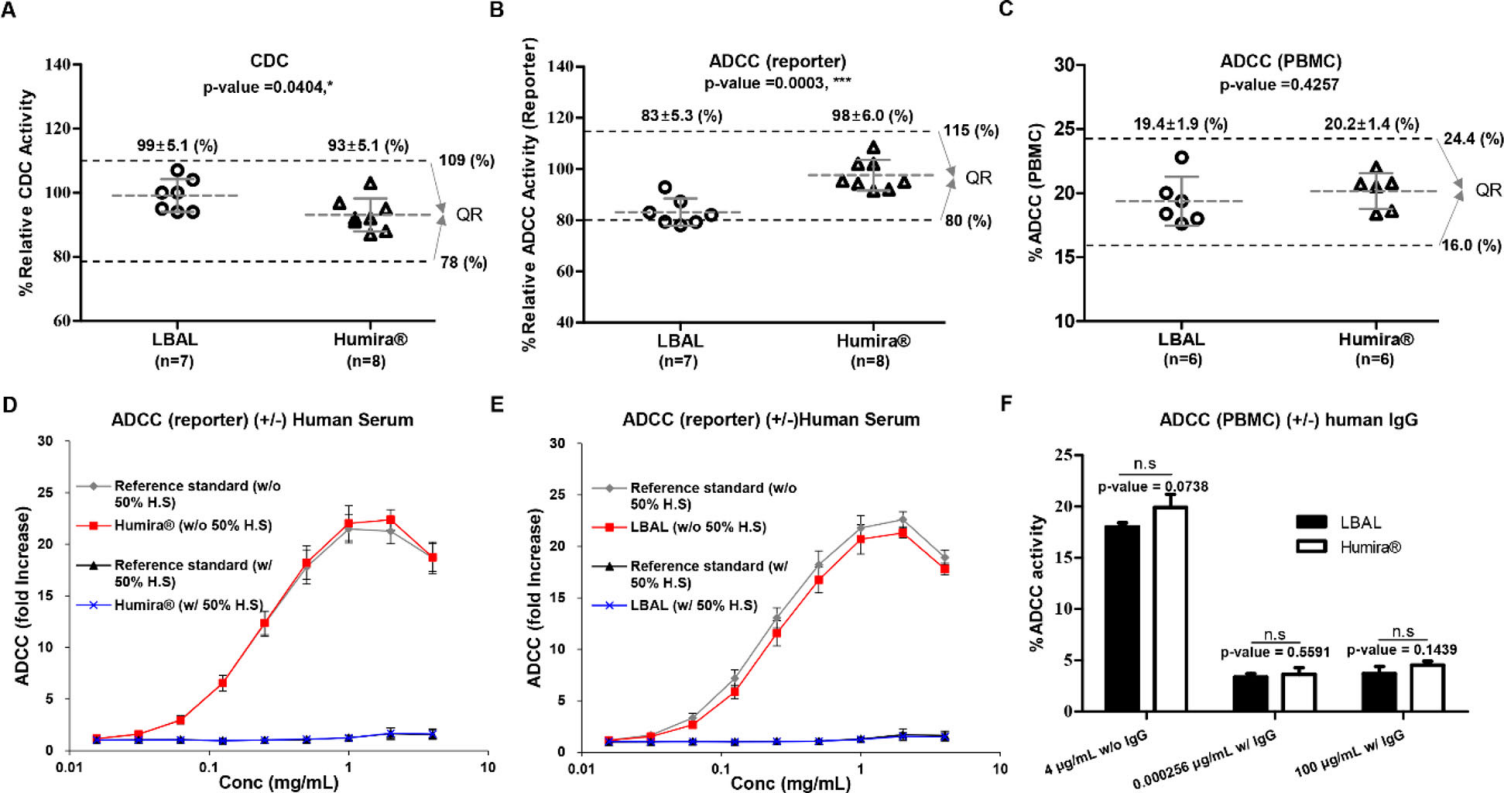
Similarity assessment of Fab-/Fc-related biological activities: comparison of Humira® and LBAL. Scatter dot plots of (**A**) CDC, (**B**) ADCC (reporter), and (**C**) %ADCC (PBMC), (**D** and **E**) ADCC (reporter) between Humira® (D) and LBAL (E) under 50% human serum (H.S) condition (±), and (**F**) ADCC (PBMC) under human IgG (10 mg/mL) condition (±). Dashed lines (black) represent the similarity quality range (QR) (mean ± 3SD). Student’s *t*-test was used for statistical analysis. *p < 0.05, **p < 0.01, ***p < 0.001, and ‘n.s’ indicates ‘not significant’, *p* ≥ 0.05.

The CDC activity was measured by the cell viability (MTS) assay using tmTNFα-CHO target cells in the presence of soluble C1q. The CDC activity of LBAL (94–107%) resembled that of Humira®, within the QR of Humira® (78–109%) (Figure 5A). Next, we compared the relative ADCC activity using both Jurkat reporter cell lines and PBMCs as effector cells together with tmTNFα-CHO target cells. In the ADCC reporter bioassay, LBAL showed a slightly lower ADCC activity than Humira®, and the mean ranges of relative ADCC activity of LBAL (78–93%) were marginally outside the QR of Humira® (80–115%) (Figure 5B). In this highly sensitive, artificial test system, the ADCC activity can be much higher than under normal physiological conditions due to overexpression of both FcγRIIIa on surrogate effector cells and tmTNF-α on target cells (Zhang et al. 2020). In contrast to the ADCC reporter assay, LBAL exhibited similar ADCC activity in an *ex vivo* ADCC assay using PBMCs (Figure 5C), which was thought to be a more appropriate test system, more closely representing *in vivo* conditions. The mean ranges of %ADCC activity of LBAL (17.6–22.8%) were within the QR of Humira® (16.0–24.4%).

To assess the significance of the slightly lower ADCC activity of LBAL observed in the reporter assay, additional experiments were conducted under conditions mimicking the physiological environment. The ADCC activity of Ab drugs contributing to their therapeutic effects *in vivo* is known to be attenuated by serum/IgG (Iida et al. 2006, 2009; Satoh et al. 2006). Thus, the ADCC activity of Ab needs to be tested in the presence of serum or IgG to estimate its therapeutic effects. The ADCC activity (reporter assay) of both Humira® and LBAL was substantially reduced to undetectable levels in the presence of human serum, unlike that in the absence of human serum (Figure 5D and 5E, respectively). Consistent with the reporter bioassay results, the ADCC activity (PBMC) was remarkably decreased to an equivalent level in the presence of human IgG (Figure 5F). These findings suggest that the ADCC activity of LBAL and Humira® is likely to be comparable under physiological conditions. Therefore, the observed difference in the ADCC reporter bioassay in the absence of human serum or IgG was not clinically meaningful. Consequently, the Fab-/Fc-related biological activities (effector function) of LBAL and Humira® were considered comparable, despite minor differences in ADCC, which are further depicted in detail (Supplementary Table S4).

## Discussion

The structural properties of a protein are key determinants for its functional characteristics. Consequently, biosimilar development relies on a comprehensive analytical characterization. LBAL was developed by LG Chem in accordance with the regulatory requirements. The current study applied a systematic analytical approach using advanced and orthogonal techniques to demonstrate the structural and functional similarities between LBAL and Humira® in terms of quality, safety, and efficacy. The results of the comprehensive similarity assessment designed to compare the physicochemical and biological quality attributes between LBAL and Humira® are summarized in Table 1. Detailed results of the biological similarity assessment are described in Supplementary Table S2–S4.

The similarity assessment evaluated critical and relevant quality attributes for both LBAL and Humira®. These attributes included the primary structure, post-translational modifications, *N*-glycan profiles, higher-order structure, disulfide linkage, and biological activities related to the known or putative MOA and therapeutic effects (Table 1).

A biosimilar should have an identical amino acid sequence to an RP as a prerequisite requirement (EMA 2014a, 2014b; US FDA 2015b). We have decisively shown that LBAL has the same primary structure as adalimumab (Humira®) based on intact molecular mass analysis and peptide mapping by LC-UV/MS. Peptide mapping data collected by LC-MS indicated an identical *N*-glycosylation site (Asn 301 on the HC) and similar N-terminal variants (N-terminal pyroglutamate), C-terminal Lys variants, and post-translational modification profiles, including Asn deamidation and Met oxidation. Full amino acid sequencing and N-/C-terminal sequencing using trypsin/Lys-C peptide mapping combined with LC-MS/MS further confirmed 100% sequence coverage (214 amino acids for LC and 451 amino acids for HC) (data not shown). The free sulfhydryl contents were comparable by the Measure-iT™ thiol assay (data not shown).

The secondary and tertiary structures of a protein are essential for their action as the three-dimensional structural motifs define the protein’s functionality. Therefore, the comparison of higher-order structures is a critical element in the similarity assessment of biosimilar products. Disulfide linkage by LC-MS, spectroscopic, and calorimetric approaches, including far-UV CD, near-UV CD, FTIR, and DSC, confirmed the highly similar secondary and tertiary structure between LBAL and Humira®.

Glycosylation of an Ab impacts effector functions, including CDC and ADCC, and pharmacokinetics by modulating the binding to C1q, FcRs (e.g. FcγRIIIa and FcRn), and mannose-binding receptors (Jefferis et al. 1998; Shields et al. 2002; Jefferis 2009; Goetze et al. 2011; Reusch and Tejada 2015) and affecting the Ab conformation and stability (Zheng et al. 2011). Thus, the Fc *N*-glycosylation profiles are regarded as a critical quality attribute among physicochemical quality attributes. *N*-glycan profiles were analyzed by HILIC-UPLC/MS. We found that neutral biantennary *N*-glycans were dominant species, and Afucose- and HM-types were minor species in LBAL and Humira®. The level of galactosylation (%) was similar, albeit there were small differences in the relative abundances of afucosylation, HM, and sialylation. LBAL contained slightly higher levels of %[Afucose] (approximately 2% difference) and %[sialylation] (<1% difference), and lower levels of %[HM] (about 6% difference) (Supplementary Table S1). We showed that LBAL and Humira® have similar binding activities for C1q, FcγRs, and FcRn and CDC, but not ADCC. The difference in %[HM] content was deemed clinically irrelevant, considering similar pharmacokinetic profiles in non-clinical/clinical studies (data not shown), which were consistent with the comparable FcRn-binding activity between LBAL and Humira®. It has been reported that the sum of %[Afucose + HM] content correlates with an increase in both FcγRIIIa-binding and ADCC activities (Lee et al. 2017; Lee et al. 2019). The relatively lower levels of %[Afucose + HM] in LBAL (around 4% difference) were thought to be reflected in the slightly lower ADCC potency seen in the *in vitro* reporter bioassay, even though a highly similar FcγRIIIa-binding activity was observed. However, this difference in the reporter assay was believed to be due to the extremely sensitive, artificial test system combined with overexpression of both FcγRIIIa on effector cells and tmTNF-α on target cells, unlike the normal physiological assay conditions (Zhang et al. 2020). In addition, there were no differences in an orthogonal assessment of ADCC activity using physiologically relevant PBMCs, which was considered a suitable test system to more closely simulate physiological conditions. Furthermore, when monoclonal Ab is used therapeutically, nearly all the cellular FcγRs are occupied with IgG due to the very high concentration of IgG (∼15 mg/mL) in serum (Nimmerjahn and Ravetch 2008). When we explored the effects of the difference in ADCC activity detected in the reporter assay under the presence of human serum or IgG, we showed that such a difference would not significantly contribute to therapeutic impacts under physiological conditions.

Lastly, highly similar biological activities were observed between LBAL and Humira® in bioassays indicative of known and potential MOA of adalimumab, with only a slight difference in ADCC potency. As Fab-related biological quality attributes, the sTNF-α/tmTNF-α-binding, TNF-α neutralization, and apoptosis (via reverse signaling) were evaluated by a similarity assessment. As Fc-related biological attributes, the binding to FcγRs (FcγRI, FcγRIIa (R/H), FcγRIIb, FcγRIIIa (V/F), and FcγRIIIb), C1q, and FcRn were investigated. Furthermore, as Fab- and Fc-related biological attributes, the effector functions, such as CDC and ADCC, were determined to demonstrate the biosimilarity of LBAL to Humira®. As mentioned in the justification for the difference in %[Afucose + HM] contents, the ADCC potency is likely to be similar between LBAL and Humira® under physiological conditions. Therefore, the observed differences in the most sensitive ADCC reporter assays under the absence of human serum or IgG were expected to be clinically irrelevant, which was supported by clinical studies (data not shown).

In summary, the analytical similarity between LBAL and its RP Humira® has been comprehensively demonstrated in compliance with the appropriate regulatory guidance. The similarity studies addressed the primary and higher-order structures, *N*-glycan profiles, and disulfide linkage as structural/physicochemical quality attributes, in addition to the Fab-, Fc-, and Fab-/Fc-mediated biological activity as biological/functional quality attributes. Some minor differences in quality attributes were detected. However, these differences have been fully examined and justified as clinically irrelevant. The findings presented here are considered a foundation to support the overall similarity in non-clinical and clinical studies of LBAL.
